# *Ex-Vivo* Perfusion of Limb Vascularized Composite Allotransplants: A Systematic Review of Published Protocols

**DOI:** 10.3389/ti.2025.14132

**Published:** 2025-05-19

**Authors:** Tessa E. Muss, Eleni M. Drivas, Amanda H. Loftin, Yinan Guo, Yichuan Zhang, Christopher D. Lopez, Alisa O. Girard, Isabel V. Lake, Bashar Hassan, Richa Kalsi, Byoung Chol Oh, Gerald Brandacher

**Affiliations:** ^1^ Department of Plastic and Reconstructive Surgery, Vascularized Composite Allotransplantation (VCA) Laboratory, Johns Hopkins University School of Medicine, Baltimore, MD, United States; ^2^ Department of Pathology, Johns Hopkins University School of Medicine, Baltimore, MD, United States; ^3^ Division of Plastic and Reconstructive Surgery, Cooper University Health Care, Camden, NJ, United States; ^4^ Division of Plastic Surgery, American University of Beirut, Beirut, Lebanon; ^5^ Department of General Surgery, University of Maryland Medical Center, Baltimore, MD, United States; ^6^ Department of Visceral, Transplant and Thoracic Surgery, Innsbruck Medical University, Innsbruck, Austria

**Keywords:** vascularized composite allotransplantation, vascularized composite allograft, composite tissue transplantation, machine perfusion, machine preservation

## Abstract

Vascularized composite allotransplantation (VCA) has revolutionized restorative surgery of devastating injuries. Unfortunately, these grafts undergo significant injury during prolonged cold ischemia and subsequent reperfusion. *Ex-vivo* machine perfusion (EVMP) is a technique that has shown significant promise in solid organ transplant, but study of its utility in VCA has been limited. A systematic review was conducted to identify preclinical publications investigating perfusion in limb VCAs. Articles published through June 2023 were screened. 29 articles met inclusion criteria, comprising 370 VCA limbs from swine, rats, canines, and humans. EVMP was conducted under normothermic (n = 6), near-normothermic (n = 11), sub-normothermic (n = 3), or hypothermic (n = 13) conditions. While each study used a unique perfusate recipe, most were based on a premade medium. Many incorporated additives, including antibiotics and red blood cells. The duration varied from 3 to over 24 h. Multiple studies showed improved or equivalent biomarkers, histology, and outcomes for normothermic or near-normothermic EVMP (n = 4) and hypothermic EVMP (n = 8) compared to static cold storage, suggesting that EVMP may be a superior storage method to SCS. While there is no definitive evidence regarding the optimal temperature, perfusate composition, or perfusion time for VCAs, each perfusion factor should be chosen and adapted based on the individual goals of the study. This review offers a summary of the current literature to serve as an accessible reference for the design of future protocols in this field.

## Graphical Abstract

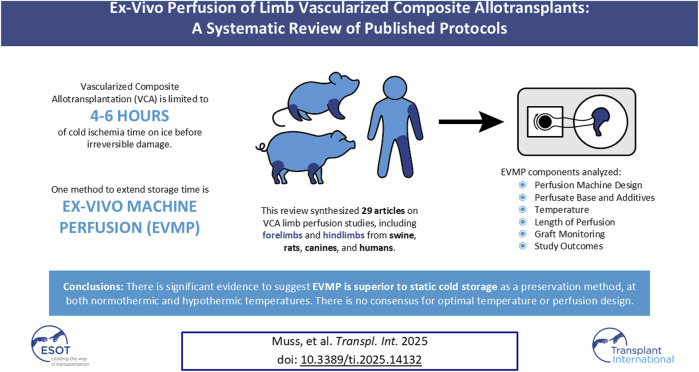

## Introduction

Vascularized composite allotransplantation (VCA) is a pioneering reconstructive approach wherein transfer of a multi-tissue allograft is used to return form and function to a site of severe tissue injury or loss [1]. In the last 25 years, more than 150 patients have undergone successful VCA, including hand, face, uterus, abdominal wall, penis, scalp, and vascularized parathyroid gland transplantation [2, 3]. Despite the life-enhancing role of VCA, these procedures carry considerable ethical and psychosocial burdens, as well as high rates of postoperative complications [4–10]. A significant challenge facing VCA is the requirement for lifelong immunosuppression and incremental allograft monitoring. While many VCAs have seen long-term success without chronic rejection, VCA procedures initially yield a disproportionate incidence of acute rejection relative to all other transplant procedures [11–16]. Graft inflammation and staged rejection are strongly influenced by allograft ischemia, temperature changes, and mechanical trauma associated with organ recovery and preservation, even under traditional static cold storage conditions [17, 18]. Interruption of allograft perfusion, and therefore cellular respiration, causes the accumulation of toxic substances and free radicals, which trigger apoptosis and tissue necrosis [19]. Sudden reperfusion increases the production of reactive oxygen species and triggers innate and adaptive immunologic responses that may impair both short- and long-term organ function [19–22]. The low ischemic tolerance of these grafts furthermore significantly limits their accessibility and utility. In response, continued advancement in VCA necessitates novel preservation strategies that decrease reperfusion injury, enhance aerobic cellular respiration, and improve outcomes.

*Ex-vivo* machine perfusion (EVMP) is an innovative technique designed to prolong preservation time and improve the function of solid organ transplants, and therefore has become an area of interest in VCA [23]. In solid organ transplantation, EVMP has enabled safe transportation while prolonging preservation time and expanding the donor pool [24]. Further, this highly modifiable system has enabled non-acceptable organs to be reconditioned for successful transplantation [25, 26]. A central asset of this technique is the ability to modify fluid pressure, flow rate, and temperature, enabling normothermic and near-normothermic tissue perfusion [27]. Independent from standard cold preservation, EVMP reduces the tissue damage and subsequent functional impairments associated with prolonged cold ischemia times and reperfusion injury [28–30]. Within the past decade, use of EVMP in animal models and solid organ transplantation has made promising strides toward improved post-transplant function and expansion of organ donor pools [30–33].

Given the disproportionate burden of tissue injury and rejection in VCA, application of EVMP has the capacity to revolutionize transplant protocols and outcomes in the field. Still, application of this technology in VCA is neoteric and nuanced. The complexities of perfusing a diversity of tissues, each with unique metabolic needs, warrant careful investigation of perfusate composition and preservation methodologies. Currently, only a modest cohort of studies have been published that document protocols and outcomes of this technique in experimental VCA models.

Despite a clear need for improved methods of VCA preservation, there is a paucity of literature evaluating successful alternative transplant perfusion protocols. The purpose of this study is to conduct a systematic review of the literature on EVMP for VCA. Specific aims include identification of all current literature on EVMP in VCA, characterization of these studies in terms of perfusion protocols, perfusate composition, monitoring, and outcomes, and comparison of these protocol attributes and outcomes to assess optimal preservation of allografts. Synthesis of results will contribute to an optimized EVMP technique in VCA and guide future research in this evolving field.

## Methods

### Literature Search

A comprehensive literature search of manuscripts listed in PubMed, Scopus, EMBASE, Cochrane Library, and ClinicalTrials.gov databases was conducted in June 2023 in compliance with Preferred Reporting Items for Systematic Reviews and Meta-Analyses (PRISMA) guidelines [34]. Titles, Abstracts, Keywords, and Mesh terms (PubMed only) were searched using the following terms: ((vascularized composite allotransplantation) OR (vascularized composite allotransplant) OR (vascularized composite allograft) OR (vascularized allograft) OR (vascularized allogeneic tissue) OR (vascularized composite tissue transplantation) OR (vascularized composite tissue transplant) OR (composite tissue allotransplantation) OR (composite tissue allotransplant) OR (composite tissue allograft) OR (composite tissue allografting) OR (composite tissue transplantation) OR (composite tissue transplant) OR (reconstructive transplant)) AND ((machine perfusion) OR (machine preservation) OR (*ex vivo* perfusion) OR (extracorporeal perfusion) OR (extracorporeal circulation)). The following filters were used in each database to fit within the inclusion criteria: “Full text” in PubMed, “Article” in Scopus, and “Article” and “Article in Press” in EMBASE. The “Trials” tab was used in Cochrane Library, and no filters were applied for ClinicalTrials.gov.

Predetermined inclusion criteria for selecting studies were [1]: preclinical articles studying normothermic, near-normothermic, sub-normothermic, and hypothermic perfusion [2]; perfusion of limbs within VCA [3]; randomized control trials, prospective and retrospective case-control and cohort studies, cross-sectional cohort studies, case reports, and technique papers. Exclusion criteria were [1]: reviews without presentation of new data [2]; abstracts, conference papers, editorials, or comments [3]; articles about solid-organ perfusion [4]; articles about non-limb perfusion; and [5] articles reporting little data on perfusion technique or outcomes.

Papers meeting exclusion criteria, duplicate publications, and articles unrelated to limb perfusion were eliminated. Remaining works were sought for retrieval as full texts, and their reference lists screened for additional relevant articles meeting inclusion criteria that were missed in the electronic search. Two independent authors (TEM and AHL) conducted the search, screening, and eligibility assessment to agree upon a comprehensive list of included articles. Controversies were resolved by discussion with a third reviewer (YG and YZ).

### Variables and Outcomes of Interest

The following variables were recorded for each included study: model species, tissue undergoing perfusion, perfusion device, perfusion temperature, perfusion flow type and rate, perfusion pressure, perfusion duration, perfusate composition (where this data was available), monitoring techniques, post-perfusion findings, and post-replant outcomes.

## Results

### Study Design

**FIGURE 1:**
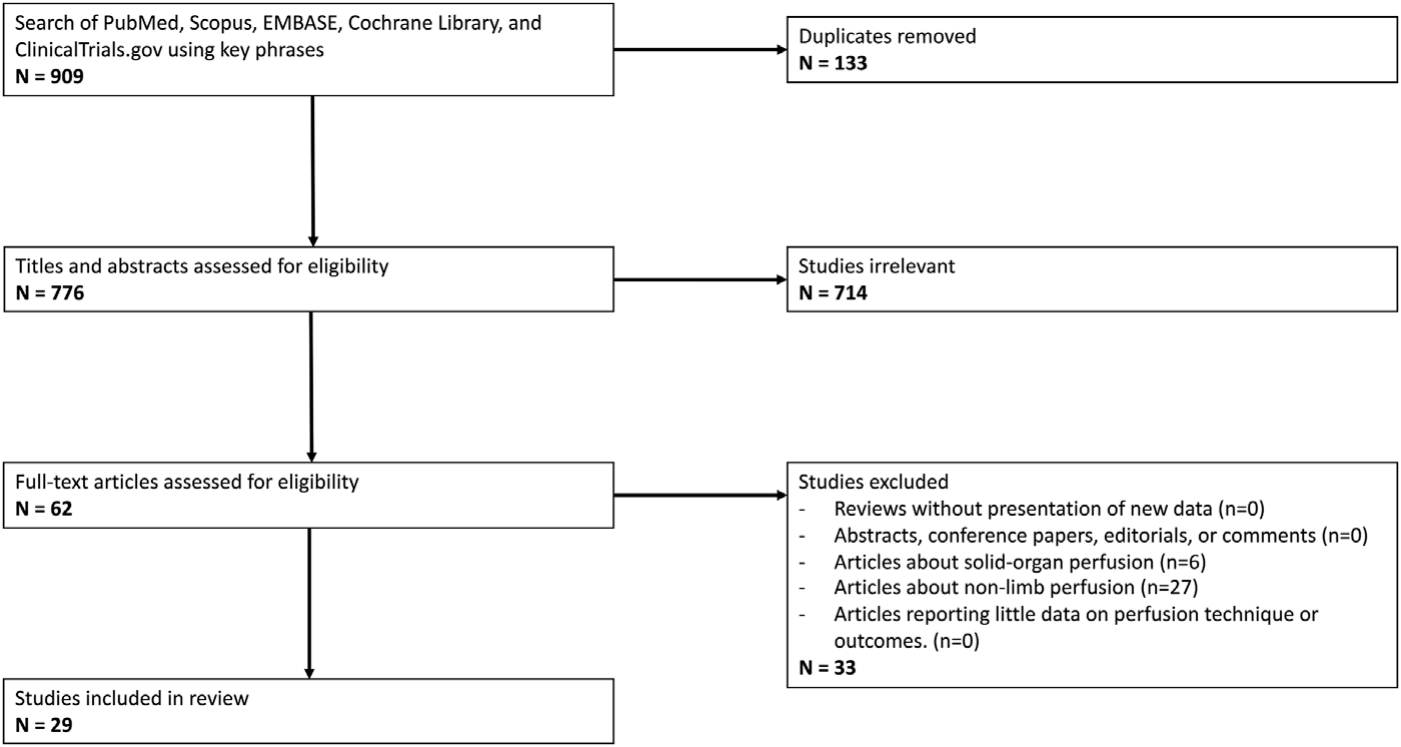
PRISMA Flow Diagram outlining inclusion and exclusion criteria, number of abstracts screened, and full texts retrieved.

Initial literature search yielded 776 unique articles, of which 29 met inclusion criteria (see Figure 1) [17, 35–62]. Despite the search terms specific to vascularized composite allotransplantation, the majority of these articles were focused on solid organ perfusion and were therefore excluded from the study. All included studies were randomized control trials published between 1985 and 2023 and cumulatively represent perfusion of 370 vascularized composite grafts (see Table 1). All grafts were limbs, of which 20 (5.4%) were human. The remainder were animal models, with the majority were harvested from swine (223, 60.3%), followed by rat (81, 21.9%) and canine (46, 12.4%). Among swine studies, 218 (97.8%) limbs were forelimbs. Eleven (36.7%) studies compared outcomes of perfused limbs against limbs placed in static cold storage. Twelve (40.0%) studies investigated outcomes after replantation (141 limbs). Most perfused grafts underwent cannulation of a single artery (335, 90.5%), although grafts perfused via two arteries were investigated by a single institution (35, 9.5%). Study comparison groups and outcomes are summarized in Table 1.

**TABLE 1:** Articles included in systematic review, n = 30.

Author (Year)	Institution	Species (details)	Limb (total #)	Cannulated arteries	Intervention (# limbs)	Comparator (# limbs)	Outcomes	Conclusion
Amin [17]	University of Manchester, UK	Swine (Landrace, 80 kg)	Fore (5)	2: brachial artery (dominant) and radial artery (collateral)	NT perfusion (5)	(0)	Cytokine concentration and leukocyte count at perfusion t = 0 and t = end (6 h)	At 6 h, there was a cumulative increase in pro-inflammatory cytokines and significant leukocyte diapedesis and depletion from the graft
Amin [35]	University of Manchester, UK	Swine (Landrace, 80 kg)	Fore (35)	2: brachial artery (dominant) and radial artery (collateral)	Experiment 1: NT at 70 mmHg (10) SNT at 70 mmHg (5) SNT at 50 mmHg (5) HT at 30 mmHg (5) Experiment 2: 2 h SCS + Optimal condition perfusion (5)	Experiment 1: Each other Experiment 2: SCS (8 h) (5)	Experiment 1: Hemodynamic and biochemical stability, to identify optimal perfusion conditions for Experiment 2 Experiment 2: Reperfusion with matched blood from unrelated donor for 4 h without immunosuppression: hemodynamic and biochemical stability	Experiment 1: NT perfusion had best outcomes and was deemed to have “optimal conditions” Experiment 2: 2 h SCS + NT perfusion was superior to 8 h SCS.
Gok [36]	UMich	Rat (275 ± 25 g)	Hind (25)	1: femoral artery or common iliac artery	NNT perfusion using: Experiment 1: Femoral artery cannulation (5) Experiment 2: Hemofilter (5) Experiment 3: 6 h NNT perfusion (5)	Experiment 1: NNT perfusion using common iliac artery cannulation (5) Experiment 2: No hemofilter (Experiment 1 limbs) Experiment 3: Contralateral limbs: No perfusion (5)	Experiment 1: Flow rate, perfusion pressure, barotrauma Experiment 2: Lactate and potassium clearance Experiment 3: Hemodynamic and biochemical stability, histology	Experiment 1: Common iliac artery cannulation offers better hemodynamics and less shear stress Experiment 2: Lactate and potassium were maintained at low levels using a hemofilter Experiment 3: Using the common iliac artery and a hemofilter, metabolic outcomes were good without barotrauma, however muscle cells were more damaged than in controls
Werner [37]	UMich	Human (3M:2F, 37–69y, BMI 22.5–43.9 kg/m^2^)	Upper (5)	1: brachial artery	NNT perfusion (5)	(0)	Hemodynamic and biochemical stability, histology, muscle contractility	Human limb allografts appeared viable after 24 h NNT perfusion
Ozer [38]	UMich	Swine	Fore (8)	1: brachial artery	NNT perfusion with autologous blood for 24 h (4)	SCS for 6 h at 4°C (4)	Hemodynamic and biochemical stability, histology; Post-perfusion transplantation to recipients (12 h monitoring)	Limb survival up to 24 h
Ozer [39]	UMich	Swine (40 ± 5 kg)	Fore (7)	1: brachial artery	NNT perfusion with autologous blood for 12 h (4)	SCS for 6 h at 4°C (3)	Hemodynamic and biochemical stability, histology; Post-perfusion transplantation to recipients (7) (12 h monitoring)	Achieved transplantation of limbs after 6 h NNT perfusion with promising contractility and biochemical stability
Constantinescu [40]	Bern University Hospital, Switzerland	Swine (Large white, 37.5 ± 5.5 kg)	Fore (16)	1: axillary artery	NNT 12 h (8)	Contralateral limbs: SCS at 4°C (8)	Hemodynamic and biochemical stability, histology	Perfused limbs demonstrated superior biochemical stability and muscle contractility compared to controls
Fahradyan [41]	Cleveland Clinic	Swine (Yorkshire, 45 kg)	Fore (20)	1: subclavian artery	12h group: NT perfusion for 12 h (5) >24h group: NT perfusion until vascular resistance increased: Systolic pressure >115 mmHg, compartment fullness, weight gain, O2 decrease by 20% (5)	Contralateral limbs: SCS at 4°C (10)	Muscle contractility, compartment pressure, tissue O2 saturation, indocyanine green angiography, thermography	Outcomes of prolonged NT perfusion (>24 h) are not significantly different from 12 h NT perfusion
Duraes [42]	Cleveland Clinic	Swine (Yorkshire, 45 kg)	Fore (36)	1: subclavian artery	NT perfusion for 12 h (18), with evolving protocol of WIT, CIT, perfusate contents, and perfusate temperature	Contralateral limbs: SCS at 4°C for 12 h (18)	Muscle contractility, compartment pressure, tissue O2 saturation, indocyanine green angiography, thermography	Perfusion preserved limb physiology and function for up to 12 h. Limbs with best outcomes: Colloid + washed RBC perfusate at 39°C for 12 h
Haug [43]	BWH	Swine (Yorkshire, 40 kg)	Fore (8)	1: axillary artery	HT perfusion for 12h, using either modified STEEN (2), balanced electrolyte Phoxilium (2), or dextran-enriched Phoxilium (PHODEX) (2)	SCS at 4°C for 12 h (2)	Hemodynamic and biochemical stability, histology, HIF1a	PHODEX is an affordable substitute for STEEN, with exception to elevated creatine kinase and lactate dehydrogenase
Haug [44]	BWH	Human (2M:1F, 24–51y, BMI 22.3–29.1 kg/m^2^)	Upper (6)	1: brachial artery	HT perfusion for 24 h (3)	Contralateral limbs: SCS for 24 h (3)	Hemodynamic and biochemical stability, histology, HIF1a	HT perfusion extended preservation time to 24 h
Kueckelhaus [45]	BWH and Germany	Swine (Yorkshire, 38.4 ± 1.5 kg)	Fore (7)	1: Unspecified	HT perfusion for 12 h using portable perfusion machine and subsequent heterotopic replantation (3)	SCS at 4°C for 4 h and subsequent heterotopic replantation (4)	Hemodynamic and biochemical stability, histology, cytokine levels	Perfused limbs were superior to SCS limbs after transplantation
Kueckelhaus [46]	BWH and Germany	Swine (Female Yorkshire, 50–60 kg)	Hind (10)	1: femoral artery	HT perfusion using portable perfusion machine (5)	SCS for 12 h (5)	Hemodynamic and biochemical stability, histology	Successful perfusion via portable device, superior to SCS.
Krezdorn [47]	BWH and Germany	Swine (Female Yorkshire, 35–45 kg)	Fore (8)	1: axillary artery	HT perfusion for 24 h and subsequent replant onto same animal (4)	SCS at 4°C for 4 h and subsequent replant onto same animal (4)	Hemodynamic and biochemical stability, histology, 7-day monitoring of animals	Perfused limbs were comparable to SCS limbs and may reduce muscle damage and systemic reactions on replantation
Krezdorn [48]	BWH	Swine (Female Yorkshire, 35–45 kg)	Fore (8)	1: axillary artery	HT perfusion at 10°C for 2 h and subsequent replantation onto same animal (3) Or HT perfusion at 10°C for 12 h and subsequent replant onto same animal (3)	SCS at 4°C for 2 h and subsequent replant onto same animal (2)	Hemodynamic and biochemical stability, histology, PCR of target genes	Perfused limbs demonstrated downregulation of genes involved in glycolysis, angiogenesis, and DNA damage compared with SCS limbs
Kruit [49]	Radboud University Medical Center, Netherlands	Swine (Female Dutch Landrace, ∼69 kg)	Fore (24)	1: brachial artery	HT perfusion for 18 h and subsequent replant onto the same animal (6)	SCS at 4°C–6°C for 4 h and subsequent replant onto the same animal (6) Sham surgery in contralateral limbs (12)	Hemodynamic and biochemical stability, histology, nerve stimulation, 12 h monitoring of animals	Muscle contraction comparable between perfused, SCS, and sham limbs, perfused limbs had greater edema than SCS limbs. There was no correlation between muscle function and histology
Domingo-Pech [50],	Spain	Canine (Mongrel)	Hind (21)	1: iliac artery	Perfusion for 24 h (9) Perfusion for 24 h and subsequent replantation onto same animal (6)	Limb harvest and immediate replant (6)	Hemodynamic and biochemical stability, histology, 6 h monitoring of animals	Edema was managed with peripheral vasodilators, steroids, and cool perfusate temperature
Usui [51]	Japan	Canine (Mongrel, 10–15 kg)	Hind (46)	1: femoral artery	Intermittent perfusion with fluorocarbon at room temp (9) or HT (6); Continuous perfusion with fluorocarbon at room temp (6) or HT (5); Continuous perfusion with Lactated Ringer’s at HT (5) All limbs were replanted	Limb harvest and immediate replantation (15)	6 h monitoring of animals	Fibrocarbon inhibited anaerobic metabolism and creatine phosphokinase leak from the limb and was more pronounced under continuous and HT perfusion conditions
Muller [52]	Bern University Hospital, Switzerland	Swine (Large white, 39 ± 5.5 kg)	Fore (61)	1: unspecified	6 h SCS/12 h perfusion (7) 12 h SCS/5 h perfusion (6) No SCS/12 h perfusion/replantation (11) 6 h SCS/12 h perfusion/replantation (8)	Contralateral limbs SCS for 18 h (10) Contralateral limb biopsies at euthanasia (19)	Hemodynamic and biochemical stability, histology, inflammatory markers, 7-day monitoring of replanted animals	No significant difference in markers for ischemia/reperfusion injury
Adil [53]	University of Toronto	Rat (Male Lewis, 300–430 g)	Hind (4)	1: femoral artery	Decellularization perfusion for 5 days (4)	(0)	Hemodynamic and biochemical stability, histology	Successful decellularization
Burlage [54]	MGH	Rat (Lewis, 250–300 g)	Hind (39)	1: femoral artery	HT perfusion with BSA for 6 h (4) HT perfusion with BSA/PEG for 6 h (4) HT perfusion with HBOC-201 for 6 h (4) HT perfusion with HBOC-201 for 6h, then transplant (13)	SCS 6h, transplant (4) SCS 24h, transplant (5) Direct transplant after harvest (5)	Hemodynamic and biochemical stability, histology	Lower edema with HBOC-201 perfusate compared to BSA and BSA/PEG, decreased energy charge ratios in SCS compared to HBOC-201
Figueroa [55]	Cleveland Clinic	Swine (Yorkshire, 45 kg)	Fore (24)	1: subclavian artery	NNT perfusion with HBOC-201 (6) NNT perfusion with RBC perfusate (6)	SCS at 4°C (12)	Hemodynamic and biochemical stability, histology	No significant differences between HBOC-201 and RBC-perfused limbs
Gok [56]	UMich	Rat (Male Lewis, 250 ± 2.5 g)	Hind (25)	1: unspecified	HT perfusion with HTK for 6h, then transplant (5)	No intervention (5) Sciatic nerve transected and directly repaired (5) Limb harvest and immediate transplant (5) HTK flush, 6h SCS, then transplant (5)	Hemodynamic and biochemical stability, histology, muscle contractility after 12 weeks	No significant differences in myocyte injury in HT perfusion group compared to controls, decreased muscle force in HT perfusion after 12 weeks compared to controls
Goutard [57]	MGH	Rat (Lewis, 250 ± 50 g)	Hind (32)	1: femoral artery	HT perfusion 3 h (4) 12h SCS, HT perfusion 3 h (4) 18h SCS, HT perfusion 3 h (4) 12h SCS, HT perfusion 3h, transplant (4)	Direct transplant (4) SCS 12–48h, transplant (16)	Hemodynamic and biochemical stability, histology, 21-day monitoring of animals	No differences in survival for 0–24 h SCS, frequent delayed graft failure for 48h SCS, increased edema in 18 h SCS perfusion compared to 12h SCS, improved clinical appearance 12 h SCS perfusion transplants compared to 12 h SCS only
Mayer [58]	Humboldt Univerty, Berlin, Germany	Swine	Fore (60)	1: unspecified	NNT perfusion (60)	(0)	Hemodynamic and biochemical stability	Viability of flaps for up to 27 h
Rezaei [59]	Cleveland Clinic	Human (Adult DBD)	Upper (20)	1: brachial artery	NT perfusion 48 h at 38°C (10)	SCS at 4°C (10)	Hemodynamic and biochemical stability, histology	Improved histology and decreased edema in perfusion compared to SCS
Stone [60]	University of Manchester, UK	Swine (Landrace, 80 kg)	Fore (10)	1: brachial artery	NT limb + kidney perfusion 5 h (5) NT limb only perfusion 5 h (5)	(0)	Hemodynamic and biochemical stability, histology, inflammatory markers, thermal imaging	Addition of a kidney rapidly stabilized lactate, bicarbonate, and pH levels, more homogenous global perfusion in kidney group compared to limb only
Taeger [61]	University Hospital Regensburg, Germany	Human (Adult traumatic amputations)	Lower (2)	1: femoral artery	HT perfusion followed by reattachment to patient (2)	(0)	3-month follow-up	Successful replantation in both patients
Valdivia [62]	Hannover Medical School, Germany	Rat (Lewis, 227–400 g)	Hind (30)	1: femoral artery	HT perfusion 4 h with lentiviral vectors (15) HT perfusion 4 h (15)	(0)	Hemodynamic and biochemical stability, histology, cytokine levels, bioluminescence detection, cell phenotyping	No significant tissue damage from lentiviral vector use

C, continuous flow; Fore, forelimbs; h, hours; Hind, hindlimbs; HT, hypothermic; N_2_, nitrogen; NR, not reported; NT, normothermic; NNT, near-normothermic, P, pulsatile flow; q#time, to indicate frequency a medication was administered; SCS, static cold storage; SNT, sub-normothermic; Upper, upper limbs.

### Perfusion Technique

Perfusion was achieved under varying temperature conditions: normothermic (NT, 38°C–39°C) in 6 studies, near-normothermic (NNT, 27°C–35°C) in 11 studies, sub-normothermic (SNT, 20°C–22°C) in 3 studies, and hypothermic (HT, 4°C–12°C) in 13 studies (see Table 2). Pump-controlled perfusate flow was pulsatile (7 studies), continuous (12 studies), or intermittent (cyclically paused and resumed, 1 study), although 9 studies provided insufficient detail to determine flow pattern. Seven studies discussed a technique to initiate perfusion, requiring up to 1 h to reach target pressure, flow, and temperature parameters. Perfusion was performed for 3–6 h (9 studies), 12 h (10 studies), 18 h (1 study), 24 h (5 studies), or longer (4 studies), with the longest perfusion achieved via normothermic pulsatile perfusion for 44 h [41]. While perfusate gas composition varied widely, all studies applied oxygen to the perfusion circuit.

**TABLE 2:** Details of perfused limbs.

Author (Year)	WIT	CIT (target)	Perfusion device	Flow type	Relative perfusate temp	Actual perfusate temperature (target) (˚C)	Perfusion initiation technique	Perfusate flow rate (% of *in vivo* baseline measurements)	Perfusion pressure (target) (mmHg)	Vascular resistance	Gas content	Perfusion duration (h) (target)
Amin [17]	25 ± 2.7 min	124.6 ± 6.2 min (120 min)	Centrifugal pump	NR	NT	37.1 ± 0.1 (38)	Pressure increase 5 mmHg Q5 min	119.8 ± 12.75 mL/min/Kg; 356 ± 131.5 mL/min	MAP: 69.5 ± 0.4; (70)	Decreased until t = 1 h, stable thereafter	95% O_2_/5% CO_2_	6
Amin [35]	NR	NR	Centrifugal pump	NR	NT NNT HT	NR (38) NR (28) NR (10)	Pressure increase 5 mmHg Q5 min	102.3 ± 34.8 mL/kg/min	MAP: 65.6 ± 6.7	NT at 70 mmHg: 0.4 ± 0.3 mmHg/min/mL, stable, uniform	95% O_2_/5% CO_2_	6
Gok [36]	NR	NR	Peristaltic roller pump (Masterflex L/S peristaltic pump	P	NNT	NR (30–35)	Flow at t = 0 0.1 mL, increased incrementally to 2.5 mL/min over first 20 min	Experiment 3: 0.9 ± 0.24 mL/min	Experiment 3: 33.74 ± 14.83	Gradual decrease	95–100% O_2_; adjusted to maintain pO_2_ 225–400 mmHg/0%–5% CO_2_	6
Werner [37]	76min	NR	Roller pump (Shiley Roller Pump)	P	NNT	32.0 ± 0.2 (30–33)	NR	310 ± 20 mL/min (6%–10%)	Systolic: 93 ± 2	0.4 ± 0.3 mmHg/min/L	40–60% O_2_/5–10% CO_2_/Remaining% N_2_	24
Ozer [38]	NR	NR	Perfusion pump (Waters Medical Systems, Minneapolis, MN)	P	NNT	NR (27–32)	NR	80 mL/h	MAP: 60–80	Increased until t = 1 h, decreased after t = 2 h	95% O_2_/5% CO_2_	24
Ozer [39]	NR	NR	RM3 pulsatile perfusion pump (Waters Medical Systems, Minneapolis, MN)	P	NNT	NR (27–32)	NR	80–120 mL/h	MAP: 60–80	High at t0 = 3 h, later normalized	95% O_2_/5% CO_2_	12
Constantinescu [40]	1 h	NR	Turbine pump (MEDOS Deltastream Blood Pump, Model DP2; Medos Medizintechnik AG, Stolberg, Germany)	C	NNT	NR (32)	NR	100–150 mL/min (50%)	MAP: 33.73 ± 2.06	NR	21% O_2_; arterial pO2 128.81 ± 8.82 mmHg	12
Fahradyan [41]	NR	NR	Roller pump (Terumo Sarns 8000) fitted with a pulse module (Terumo Sarns)	P	NT	NR (38)	Flow and temp were gradually increased during first hour	12 h group: 0.77 ± 0.1 L/min >24 h group: 0.43 ± 0.03 L/min	12 h group: Systolic: 107.25 ± 31.02 Diastolic: 44.69 ± 21.10 >24 h group: Systolic: 111.14 ± 12.48 Diastolic: 64.25 ± 14.15	12 h group: +6.4% ± 18.4% >24 h group: +33.3% ± 23.6%	12 h group: 100% O_2_/7% CO_2_/93% N_2_ >24 h group: 100% O_2_ 1 L/min	12 h group: 12 >24 h group: 24–44
Duraes [42]	12h 39°C colloid/wRBC: 112 ± 68 min	12h 39°C colloid/wRBC: None	Roller pump (Terumo Sarns 8000) fitted with a pulse module (Terumo Sarns)	P	NNT NT	n = 1 (N/A) n = 7 (32) n = 8 (39)	NR	NR	NR	NR	100% O_2_ + 7% CO_2_/93% N_2_	6–12 (12)
Haug [43]	77.5 ± 5.24 min	NR	Peristaltic machine pump (Master Flex Pump L/S, Cole-Parmer, Illinois, USA)	C	HT	(10)	NR	20 mL/min	24.48 ± 10.72	NR	377.22 ± 89.58 mmHg	12
Haug [44]	Median: 90min (65–155 min)	Median: 67 min (37–148 min)	Peristaltic machine pump (Master Flex Pump L/S, Cole-Parmer, IL)	C	HT	Median: 9.43 (Range 4.8–14.3) (10)	NR	Median: 30.4 mL/min	30	NR	385.4–609.7 mmHg, median 555.8 mmHg	24
Kueckelhaus [45]	NR	NR	NR	C	HT	10 ± 1.9 (10)	NR	NR	30	NR	Oxygenator used	12
Kueckelhaus [46]	NR	NR	Peristaltic pump	C	HT	(10–12)	NR	NR	30	NR	Oxygenator used	12
Krezdorn [47]	26.2 ± 14.4 min	NR	Pump	C	HT	(8)	NR	Fluctuating	29.4 ± 0.6		8.2 ± 0.7mL/100 mL	24
Krezdorn [48]	NR	NR	NR	NR	HT	(10)	NR	NR	NR	NR	Oxygenated	12
Kruit [49]	NR	NR	Centrofugal pump (BP-50 Bio-Pump Centrifugal Blood Pump, Medtronic)	NR	HT	(8–10)	NR	16 ± 1.7 mL/min	<30	NR	95% O_2_/5%CO_2_	18
Domingo-Pech [50]	NR	NR	Sarns low velocity pump	NR	HT	“Cold”	NR	NR	>100	NR	Oxygenated	24
Usui [51]	NR	NR	NR	C or I	SNT HT	(∼20) (4)	NR	NR	50	NR	Oxygenated, >400 mmHg in Fluorocarbon group	C: 6 h I: 20min perfusion for 3 or 5 cycles
Muller [52]	NR	Group 1: 6.2 ± 0.03 h (6) Group 2: 12.9 ± 1.5 h (12) Group 4: 6.2 ± 0.2 h (6)	MEDOS DataStream blood pump, model DP2 (Medos Medizintechnik AG, Germany)	NR	NNT	(32)	NR	100–150 mL/min	NR	NR	Oxygenated	Group 1: 12.1 ± 0.2 (12) Group 2: 4.9 ± 1.9 Group 3: 12.0 ± 0.3 (12) Group 4: 12.0 ± 0.1 (12)
Adil [53]	NR	NR	Peristaltic pump	C	NT	NR	NR	1 mL/min	NR	NR	NR	120
Burlage [54]	10–15 min	NR	Rotating pump (Drive Mflex L/S, Cole-Parmer, IL)	C	HT	NR	NR	HBOC-201: median 0.4 mL/min	30–40	Decreased within 1st hour, stable afterwards	Oxygenated	6
Figueroa [55]	HBOC-201: 35.50 ± 8.62 min RBC: 30.17 ± 8.03 min	NR	Roller pump (Terumo Sarns 8000)	C	NNT	HBOC-201: 33.23 ± 1.11 RBC: 33.12 ± 1.69 (38)	Temperature raised from 27°C to 38°C over 1 h	HBOC-201: 325 ± 25.00 mL/min RBC: 444.73 ± 50.60 mL/min	HBOC-201: 78.50 ± 10.75 RBC: 85.70 ± 19.90 (MAP 90)	HBOC-201: 214.80 ± 69.80 mmHg/min RBC: 190.90 ± 58.33 mmHg/min	Oxygenated	HBOC-201: 22.50 ± 1.71 RBC: 28.17 ± 7.34
Gok [56]	30 min avg	NR	Peristaltic roller pump (Masterflex L/S peristalitic pump, Cole-Palmer, IL)	C	HT	(10–15)	NR	NR	20–40	NR	Oxygenated	6
Goutard [57]	NR	NR	Roller pump (Drive Mflex L/S, Cole-Palmer, IL)	C	SNT	(21)	NR	0.8 mL/min	30–50	Decreased over 3 h	Oxygenated	3
Mayer [58]	NR	NR	NR	NR	NNT	NR	NR	NR	NR	NR	Oxygenated	NR
Rezaei [59]	59.6 ± 20.9 min	NR	Roller pump	C	NNT	35.1 ± 1.7 (38)	Flow gradually increased over 1 h	0.41 ± 0.06 L/min	MAP 90	187.3 ± 26.7 mmHg x min/L	Humidified 100% O_2_	41.6 ± 9.4 h (48)
Stone [60]	20.6 ± 3.0 min	195.4 ± 13.7 min (180 min)	NR	NR	NT	(38)	Pressure 55 mmHg, increased by 5 every 5min to reach target, limbs added after 1 h renal perfusion	Limb/Kidney: 496 ± 78.29 mL/min Limb Only: 232 ± 106.6 mL/min	75	NR	95% O_2_, 5% CO_2_	5
Taeger [61]	NR	NR	ECMO Pediatric Set (Quadrox, Maquet, Germany)	P	SNT	(20)	NR	NR	NR	NR	100% O_2_	Patient 1: 15 h 49 min Patient 2: 12 h 27 min
Valdivia [62]	NR	NR	NR	NR	HT	NR	NR	NR	NR	NR	Oxygenated	4

CIT, Cold ischemia time, time in cold storage. I, intermittent; WIT, Warm ischemia time, time from limb procurement until cold flush. Numbers formatted as mean ± standard deviation (SD).

### Perfusate Composition

Among the studies, 29 unique perfusate recipes were used and four studies experimented with different perfusate recipes (see Table 3). Twenty studies (69.0%) used a premade medium, including STEEN (6 studies), Perfadex (3 studies), Ringer’s solution (3 studies), Lactated Ringer’s solution (3 studies), Custodiol HTK (2 studies), Phoxilium (1 study), Dulbecco’s Modified Eagle’s Medium (1 study), University of Wisconsin solution (1 study), Fluosol-43 (1 study), PromoCell skeletal muscle cell growth medium (1 study), and HAM’s solution (1 study). (see Table 4). Seventeen studies (58.6%) incorporated antibiotics into the perfusate, including Cefazolin (4 studies), Vancomycin (4 studies), Meropenem (3 studies), Penicillin-streptomycin (3 studies), Piperacillin-Tazobactam (2 studies), and unnamed coverage for skin flora (1 study). One study added antifungal coverage with Amphotericin B [58], and another study wrapped the limb in an antiseptic-diluted sodium hypochlorite solution dressing for the duration of perfusion [38]. Fourteen studies (48.3%) included either red blood cells or whole blood in the perfusate, whereas the remaining 15 studies (51.7%) used acellular perfusate. Common yet inconsistently used additives were metabolic carbohydrates (e.g., glucose, dextrose, dextran; 20 studies), buffer (e.g., sodium bicarbonate, trometamol, potassium dihydrogen phosphate; 20 studies), steroids (e.g., methylprednisolone, hydrocortisone, dexamethasone; 19 studies), heparin (19 studies), insulin (17 studies), calcium (15 studies), and albumin (15 studies). Many protocols included either continuous (4, 13.8%) or periodic (12, 41.4%) partial plasma exchange, with a maximum of 13 exchanges [41].

**TABLE 3:** Perfusate content.

Author (date)	TCV	Base Medium	pRBCs	Albumin	Heparin	Antibiotics	Glucose	Buffer	Methyl-prednisolone	Insulin	Calcium	Other Additives or Notes	Perfusate exchange (volume and freq)
Amin [17]	1.1 L	500 mL Ringer’s	pRBCs: 500 mL Hct: 20%	BSA	5000 iU	500 mg meropenem	15% 10 mL/h	8.4% NaHCO_3_ 10 mL/h	500 mg	Actrapid, 10 mL/h, C	Calcium in Nutriflex, CaCl_2_ in Ringer’s	Nutriflex 10 mL/h, continuous infusion	X
Amin [35]	1.1 L	500 mL Ringer’s	pRBCs: 500 mL Hct: 20%	BSA	5000 iU	500 mg meropenem	15% 10 mL/h	8.4% NaHCO_3_ 10 mL/h	500 mg	Actrapid, 10 mL/h, C	Calcium in Nutriflex, CaCl_2_ in Ringer’s	Nutriflex 10 mL/h, continuous infusion	X
Gok [36]	>25 mL	STEEN (25 mL)	Swine RBCs to Hb 6–9 g/dL	X	2000 U	5 mg cefazolin	Glucose in STEEN	NaHCO_3_ given in 0.5-1 m Eq increments to maintain >5.0 mmol/L	10 mg	X	15 mg calcium gluconate	X	Continuous plasma filtration at 6 mL/h, replaced with equal volume Plasma-Lyte A, with 30 m Eq/L NaHCO_3_ and 1000 U/L heparin
Werner [37]	250–300 mL	X	Hb 4–6 g/dL	Albumin	Sodium heparin	Yes; skin flora coverage	Dextrose given to maintain >100 mg/dL	NaHCO_3_	200 mg at t = 0 and with each PPE	Regular insulin given if glucose >300 mg/dL	CaCl_2_	Tromethamine	PPE q3-5 h
Ozer [38]	NR	X	1:2 pRBCs:plasma, 20–30 mmHg colloid pressure	X	10,000 U	Limb in antiseptic-diluted sodium hypochlorite solution dressing	Dextran; 1 mL D_50_ given if < 4.5 mmol/L, Q2h	X	X	2U given if glucose >14 mmol/L, Q2h	X	Leukocyte and platelet fractions removed	Continuous PPE at 80 mL/h
Ozer [39]	300 mL	Dulbecco’s Modified Eagle’s Medium (200 mL)	Hct 10%	X	10,000 U	X	Dextrose in DMEM, 1 mL D_50_ given if < 4.5 mmol/L, Q2h	NaHCO3 in DMEM	X	2U given if glucose >14 mmol/L, Q2h	CaCl_2_ in DMEM	Leukocyte and platelet fractions removed	PPE 160 mL q2h (fresh contained 10% hemoglobin with similar parameters of plasma oncotic pressure)
Constantinescu [40]	NR	X	Hb 5 mg/mL	X	10,000 U	X	20 mL 10% glucose given if potassium >5.5 mmol/L	X	40 mg	15IU Actrapid given if potassium >5.5 mmol/L	X	Circuit primed with 250 mL colloid solution	X
Fahradyan [41]	2.5 L	X	Washed RBCs, Hct 10%–15%	Albumin	10,000 U	500 mg vancomycin	Maintenance D_50_	NaHCO_3_ (12 h group) or THAM (>24 h group) given if pH < 7.1	500 mg	Regular insulin 1U/h	X	X	400 mL PPE at t = 6 h and q3h thereafter
Duraes [42]	>0.5 L	X	Whole blood, Washed RBCs (Hct 10%–15%) or none	Albumin	10,000 U	500 mg vancomycin	Glucose	THAM to correct base deficit	500 mg	Regular insulin 1U/h	X	X	500 mL PPE q3h
Haug [43]	NR	(1) Modified STEEN, (2) balanced electrolyte Phoxilium, or (3) Phoxilium enriched with dextran (PHODEX)	Acellular	HSA in STEEN	2,500 U/L	X	Glucose in STEEN, 0.1% D_50_	NaHCO_3_ in STEEN and Phoxilium	125 mg	0.0075% Insulin R	CaCl_2_ in STEEN and Phoxilium	X	PPE at t = 1 h and t = 6 h
Haug [44]	NR	STEEN (XVIVO Perfusion AB, Göteborg, Sweden)	Acellular	HSA in STEEN	2,500 U/L	X	Glucose in STEEN, 0.1% D_50_	NaHCO_3_ in STEEN	125 mg	0.0075% Insulin R	CaCl_2_ in STEEN	X	PPE at 1, 6, 12, and 18 h
Kueckelhaus [45]	5.6 L	5.6 L Perfadex (XVIVO Perfusion AB, Göteborg, Sweden)	Acellular	X	X	X	D_40_ in Perfadex	4 mL THAM at t = 0	500 mg at t = 0	30U at t = 0	X	X	4 mLD_50_, 30 U insulin, and 500 mg methylprednisolone were replenished at 7 h
Kueckelhaus [46]	5.6 L	5.6 L Perfadex (Vitrolife, Göteborg, Sweden)	Acellular	X	X	X	D_40_ in Perfadex	4 mL THAM at t = 0	500 mg at t = 0	30U at t = 0	X	X	4 mL D_50_, 30 U insulin, and 500 mg methylprednisolone were replenished at 7 h
Krezdorn [47]	4 L	4 L STEEN (XVIVO Perfusion AB, Göteborg, Sweden)	Acellular	HSA in STEEN	X	X	Glucose in STEEN, 4 mL D_50_	NaHCO_3_ in STEEN	500 mg	0.3 mL	CaCl_2_ in STEEN	X	PPE at 1, 6, 12, and 18 h
Krezdorn [48]	NR	5.6 L “Modified” Perfadex (XVIVO Perfusion AB, Göteborg, Sweden)	Acellular	X	X	X	D_40_ in Perfadex	KH_2_PO_4_ in Perfadex	X	X	X	X	X
Kruit [49]	1 L	1 L University of Wisconsin solution	Acellular	X	X	X	X	KH_2_PO_4_ in UW	40 mg	X	X	X	X
Domingo-Pech [50]	727 mL	27.5% Lactated Ringer’s solution	27.5% preserved whole blood	X	3 mg/kg initial dose	Piperacillin	20.6% Rheomacrodex (LMW dextran)	10.3% NaHCO_3_	Prednisolone	X	CaCl_2_ in LR	13.7% Mannitol	PPE q4h with addition of sodium heparin 5%, 3 mg/kg, Prednisolone 20mg, Piperacillin Na 1g, Nitroglycerin 5 mg
Usui [51]	NR	(1) Fluorocarbon (Fluosol-43) diluted in Lactated Ringer’s or (2) Lactated Ringer’s alone	Acellular	X	X	X	Glucose in Fluosol-43	NaHCO_3_ in Fluosol-43	X	X	CaCl_2_ in Fluosol-43 and LR	X	X
Muller [52]	NR	Heparinized autologous blood	NR	X	Heparinized	X	X	X	X	X	X	Initially flushed with synthetic colloid hydroxyethyl starch solution	X
Adil [53]	5 L	Sodium dodecyl sulfate	Acellular	X	X	X	X	X	X	X	X	X	X
Burlage [54]	500 mL	PromoCell skeletal muscle cell growth medium or HBOC-201	Acellular	10 g BSA	1 mL heparin	2 mL penicillin-streptomycin	X	X	100 μL hydrocortisone, 8 μg dexamethasone	100 μL	X	5 mL L-glutamine	X
Figueroa [55]	2500 mL	HBOC-201 or washed RBC	Hct 10%–15%	800 mL	5000U/L	500 mg vancomycin	X	X	500 mg	1U/L	2300 mg calcium gluconate	X	400 mL exchange every 3 h starting at 6 h
Gok [56]	NR	Custodiol HTK	Acellular	2.5 g	1000U	5 mg Cefazolin	X	NaHCO_3_ 1mEq	X	X	X	X	Hemofiltration 0.1–0.3 mL/min
Goutard [57]	200 mL	Modified STEEN solution	Acellular	BSA	2000U/L	4 mL/L Penicillin-streptomycin	Glucose in STEEN	NaHCO_3_ in STEEN	16 mg/L dexamethasone, 200 mg/L hydrocortisone	20U/L	CaCl_2_ in STEEN	X	X
Mayer [58]	NR	HAM’s solution	Acellular	BSA	X	Penicillin-streptomycin, amphotericin B	Glucose in HAM’s solution	X	X	X	CaCl_2_ in HAM’s solution	L-glutamine	Hemofiltration
Rezaei [59]	2500 mL	pRBC, FFP	1200mL pRBC, 900 mL FFP	350 mL 25% albumin	5000U	250 mg vancomycin, 250 mg cefazolin	X	X	500 mg	As needed	X	X	500 mL every 3 h starting at 6 h
Stone [60]	NR	Ringer’s solution	Hct 25%–30%	BSA	4000U	500 mg meropenem	30 mL 15% glucose	50 mL NaHCO_3_	13.2 mg dexamethasone	X	CaCl_2_ in LR	40 mL 20% mannitol GTN infusion 10 mL/hr Nutriflex infusion 10 mL/hr	X
Taeger [61]	42–50 L	Heparinized Custodiol HTK or lactated Ringer’s	Erythrocyte concentrates	X	Heparinized	4g –0.5 g piperacillin-tazobactam every 3 h	X	X	X	X	CaCl_2_ in LR	X	X
Valdivia [62]	NR	STEEN solution and Sterofundin ISO	Acellular	HSA in STEEN	X	500 μL/mL cefazolin	Glucose in STEEN	NaHCO_3_ in STEEN	X	X	CaCl_2_ in STEEN	0.06% sodium hydrogen carbonate	X

BSA, bovine serum albumin; D_50_, Dextrose 50% in normal saline; HSA, human serum albumin; MW, molecular weight; NaHCO_3_, sodium bicarbonate; NR, not reported; pRBCs, packed red blood cells; PPE, partial perfusate exchange; q#time, to indicate frequency a medication was administered; TCV, total circulating volume; THAM, trometamol or tris-hydroxymethyl aminomethane; X, not used or tested.

**TABLE 4:** Contents of base media used in perfusate preparation.

Base Medium	Contents
STEEN	Albumin, D_40_, glucose, KCl, NaCl, CaCl_2_, MgCl_2_, NaH_2_PO_4_, NaHCO_3_, NaOH
Perfadex	D_40_, NaCl, KCl, MgS, Na_2_HPO_4_, KH_2_PO_4_, glucose monohydrate
Phoxilium	CaCl_2_, MgCl_2_, NaCl, NaHCO_3_, KCl, Na_2_HPO_4_
Dulbecco’s Modified Eagle’s Medium	Amino acids, vitamins, CaCl_2_, Fe(NO_3_)_3_, MgSO_4_, KCl, NaHCO_3_, NaCl, NaH_2_PO_4_, dextrose
University of Wisconsin (UW) solution	Potassium lactobionate, KH_2_PO_4_, MgSO_4_, raffinose, adenosine, glutathione, allopurinol, hydroxyethyl starch
Fluosol-43	FC-43, Pluronic F-68, NaCl, KCl, CaCl_2_, MgCl_2_, NaHCO_3_, glucose, hydroxyethyl starch
Lactated Ringer’s solution	NaCl, KCl, CaCl_2_, sodium lactate
Ringer’s solution	NaCl, KCl, CaCl_2_, NaHCO_3_, +/- other minerals
Custodiol HTK	NaCl, KCl, MgCl_2_, CaCl_2_, histidine, tryptophan, mannitol, potassium hydrogen 2-ketoglutarate
PromoCell skeletal muscle cell growth medium	Amino acids, vitamins, fetal calf serum, fetuin, EGF, bFGF, insulin, dexamethasone
HAM’s solution	Amino acids, vitamins, glucose, NaCl, KCl, CaCl_2_, MgCl_2_, CuSO_4_, FeSO_4_, Na_3_PO_4_, ZnSO_4_

### Graft and Perfusate Monitoring

During perfusion, grafts were often monitored via capillary refill, skin or muscle temperature, skin color, neuromuscular electrical stimulation, and compartment pressure (see Table 5). All but three studies used sequential tissue samples for histological staining, single-muscle fiber contractility testing, TUNEL apoptosis assay, and/or quantification of various markers of ischemia-reperfusion injury and hypoxia. Change in graft weight during perfusion was noted in 20 studies. Perfusate levels of potassium, lactate, myoglobin, and creatine kinase were monitored and reported in 20, 20, 9, and 6 studies, respectively.

**TABLE 5:** Limb monitoring and common outcome measurements.

Author (date)	Limb monitoring	Graft weight	Potassium (mmol/L)	Lactate (mmol/L)	CK (U/L)	Mb (ng/mL)
Amin [17]	- Capillary refill, skin temp, and color Q15-60 min - Samples: Skin, muscle, vessel, at t < 0, t = end	X	6.3 ± 0.5	X	X	X
Amin [35]	- Capillary refill, skin temp, and color Q15-60min - Samples: Skin, muscle, vessel, at t < 0, t = end	NT at 70 mmHg: −0.3% ± 1.7%	NT at 70 mmHg: 7.0 ± 1.7	NT at 70 mmHg: 15.1 ± 4.8	X	X
Gok [36]	- Samples: 100 mg gastrocnemius sample, flash frozen, and stored at −80°C - Metabolomics profiling	+3.1% ± 0.4%	Increased; 6.3 ± 1.2	Increased; 4.3 ± 1.3	X	X
Werner [37]	- Palm skin temp Qh - Median and ulnar nerve electrical stimulation Q2h - Samples: Flexor carpi radialis samples at 0, 12, and 24 h of perfusion	−0.4% (−7%-+7%)	Varied, 3.0–5.5	Steadily increased from 5 to 15	X	43 K at t = 0; 92 K at t = 24 h
Ozer [38]	- Capillary refill - Skin temp - Functional electostimulation Qh; Single-fiber contractility testing - Samples: Muscle biopsies, 10 mm x 5 mm	+20% after perfusion; decreased to +15% after transplantation	Stable, no change after transplantation	Increased steadily during perfusion, no change after transplantation	X	X
Ozer [39]	- Functional electostimulation Qh; Single-fiber contractility testing - Samples: Muscle biopsies, 10 mm x 5 mm	Significant gain after perfusion; No significant gain after transplantation	Stable	Gradual increase during perfusion, normalized after transplantation	X	X
Constantinescu [40]	- Capillary refill Qh - Electrical stimulation of 3 proximal nerve bundles - Skin and muscle color Qh; Compartment pressure - Samples: Muscle, nerve, vessel biopsies at t = end; Immunofluorescence staining	Maximum of +1.32%	4.27 ± 1.38	16.83 ± 2.46	X	X
Fahradyan [41]	- Peripheral perfusion via ICG angiography, t = end - Muscle surface temp Qh - Muscle and motor nerve electrical stimulation and contractility - Flexor and extensor compartment pressure - Samples: Muscle biopsies	12 h group: −1.28% ± 8.59% >24 h group: +7.28% ± 15.05%	12 h group: 5.7 ± 1.7 >24 h group: 6.5 ± 1.8	12 h group: 9.2 ± 4.4 >24 h group: 9.6 ± 4.7	12h group: 53 K ± 15 K >24 h group: 64 K ± 32 K	12 h group: 875 ± 294 >24 h group: 1134 ± 538
Duraes [42]	- Peripheral perfusion, ICG angiography, t = end - Muscle temp - Muscle and motor nerve electrical stimulation and contractility - Flexor and extensor compartment pressure, Tissue O_2_ sat - Samples: Muscle, skin, and nerve biopsies collected at 0 and 12 h	12 h 39°C colloid/wRBC: +0.54% ± 7.35%	12 h 39°C colloid/wRBC: 5.4 ± 1.1	12 h 39°C colloid/wRBC: 9.4 ± 2.4	12h 39°C colloid/wRBC: 53 K ± 15 K	12 h 39°C colloid/wRBC: 875 ± 291.4
Haug [43]	- Samples: Muscle biopsies, hematoxylin/eosin stain, HIF-1α Western blot	SCS: +3% STEEN: +25% Phoxilium: +58% PHODEX: +36%	Decreased during first 1–2 h, increased to 6 h, stable to 12 h	Decreased during first 1–2h, increased to 6h, stable to 12 h	STEEN: +1.2 K Phoxilium: +1.5 K PHODEX: +5.5 K	STEEN: +1 Phoxilium: +121 PHODEX: +140
Haug [44]	- Samples: Muscle biopsies, HIF-1α Western blot - Cytokine analysis with ELISA	SCS: +1.4% Perfusion: +4.3%	9.6 (0 h) 5.77 (24 h)	6.9 (0 h) 2.8 (24 h)	1.4 K (0 h) 4 K (24 h)	4.4 K (0 h) 9 K (24 h)
Kueckelhaus [45]	- Samples: Muscle biopsy, histology, TEM - PCR quantification of hypoxia/ischemia markers, cytokine assay	SCS: None Perfusion: +10% ± 2%	Peaked at 3 h perfusion; SCS>perfusion after transplant	Perfusion: Increased steadily to 2.43 mM	X	Peaked at 3 h perfusion; SCS>perfusion after transplant
Kueckelhaus [46]	- Samples: Muscle biopsy, histology	SCS: None Perfusion: +44.06%	5.73 (0 h) 9.35 (12 h)	X	X	X
Krezdorn [47]	- ATP and glycogen assay - 3-Tesla MRI of muscle changes - Samples: Muscle biopsy histology	+40%	Increased during perfusion; decreased after replantation	Increased during perfusion; increased in 3 h after replantation	X	Increased during perfusion; decreased after replantation
Krezdorn [48]	- Samples: Muscle biopsies after replant, histology - PCR of genes involved in glycolysis, angiogenesis, and DNA damage	X	X	X	X	X
Kruit [49]	- Muscle core temp - Nerve stimulation, muscle contractility - Samples: Flexor and extensor muscle histology	SCS: +1.6% Perfusion: −2.7%	SCS and perfused limb potassium increased after replantation (P = 0.4), remained wnl	0.7 (18 h), remained low throughout perfusion, similar to SCS (P = 0.4)	15.6 K (18 h), higher in perfused group than SCS after replantation (P < 0.01)	X
Domingo-Pech [50],	- Samples: Muscle biopsy, H&E stain	+20–50%	X	X	X	X
Usui [51]	- *In vivo* basic metabolic panel, enzymes	Continuous perfusion with fluorocarbon: −3.4% ± 1.2% after perfusion; +26.8 ± 2.7 after replant	After replant: Immediate marked increase, stable after 30 min	After replant: Immediate marked increase, normal at 6 h only in continuous perfusion with fluorocarbon group	X	X
Muller [52]	- Samples: muscle biopsy, peripheral nerve biopsy, blood vessel biopsy, histology - Inflammatory markers, serum complement activity	X	X	X	X	X
Adil [53]	- Samples: muscle, nerve, bone, skin, vessels	X	X	X	X	X
Burlage [54]	- Samples: muscle biopsy	HBOC-201: +4.9 g BSA: +48.8 g BSA/PEG: +27.3 g	HBOC-201: 5.8 after 1 h BSA: 1.8 after 1 h BSA/PEG: 4.4 after 1 h Initially increased, stabilized after 3 h	X	X	X
Figueroa [55]	- Samples: muscle biopsy - ICG angiography - Compartment pressures	HBOC-201: +23.10% ± 3.00% RBC: +13.18% ± 22.70%	HBOC-201: 6.45 ± 1.69 RBC: 6.78 ± 1.94	HBOC-201: 14.66 ± 4.26 RBC: 13.11 ± 6.68	X	X
Gok [56]	- Samples: muscle biopsy - Muscle contractility	+3.5%	X	<2	X	X
Goutard [57]	- Samples: skin, muscle	X	Decreased over 3 h	Decreased over 3 h	X	X
Mayer [58]	X	X	X	X	X	X
Rezaei [59]	- Samples: muscle biopsy Muscle and nerve functionality - Compartment pressures	+0.4% ± 12.2%	7.6 ± 0.9	20 at median time point 15 h	956 within 1 h, 49020 at endpoint	5370 initially, 34730 at endpoint
Stone [60]	- Samples: muscle, skin, vessel - Thermal imaging	X	X	Limb/Kidney: 10.9 ± 3.5 after 1h, 7.5 ± 1.7 at endpoint Limb Only: 14.6 ± 2.2 after 1 h, 13.8 ± 3.7 at endpoint	X	X
Taeger [61]	X	X	X	X	X	X
Valdivia [62]	- Samples: skin, muscle, vessels - Bioluminescence detection - Cell phenotyping	X	X	Vector: 562.3 ± 38.9 μM Non-Vector: 577 ± 26.8 μM	X	Vector: 224.9 ± 10.3 ng/mL Non-Vector: 222.9 ± 44.8 ng/mL

C, continuous monitoring; CK, creatine kinase; Hb, hemoglobin; ICG, indocyanine green; Mb, myoglobin; PPE, partial perfusate exchange; TEM, transmission electron microscopy; wnl, within normal limits.

### Perfusion Outcomes

While the designs and objectives varied between studies, multiple studies showed improved biomarkers, histology, and outcomes for EVMP limbs compared to static cold storage (SCS) at 4°C. Four studies [35, 40, 52, 59] showed equivalent or improved outcomes in NT or NNT EVMP compared to SCS, of which one involved transplantation [52]. Eight studies [44–49, 56, 57] showed equivalent or improved outcomes in HT EVMP compared to SCS, including six which involved transplantation [45, 47–49, 56, 57].

### Human Limb Studies

Of note, four articles [37, 44, 59, 61] utilized human limbs for machine perfusion studies. Three studies [37, 44, 59] looked at upper limbs, all of which showed hemodynamically stable perfusions up to 24 h, with improved histology as compared to SCS in one study. The fourth human limb study [61] looked at traumatic lower extremity amputations; lower limbs were perfused for 12–15 h at SNT temperatures, with successful replantation in both cases.

## Discussion

EVMP is an innovative and evolving approach to solid organ preservation and reconditioning for transplantation, with great potential for clinical application to VCA. The current literature in VCA EVMP is focused mainly on upper or lower extremities, but is expanding to include a variety of perfusion protocols and subsequent structural and immunological outcomes.

### Cellular Composition of Perfusate

In transplantation, perfusion media plays a crucial role in maintaining the viability and function of the graft. These media can broadly be categorized into two types: cellular and acellular. Despite both being designed to preserve the organ, their composition and mechanisms vary significantly.

Cellular media often incorporate contents like red blood cells (RBC) or hemoglobin-based oxygen carriers which facilitate the transport of oxygen to the tissue. The inclusion of cellular components aims to create an environment that is similar to *in vivo* conditions, which may especially benefit organs or tissues with high metabolic rates. The presence of cellular elements can also enhance oxygen transport and provide essential nutrients, thereby reducing ischemic injury. Werner and Ozer both adopt cellular media and show its efficacy in preserving the viability of human and swine limbs for up to 24 h [37, 38]. However, cellular media may pose challenges such as inflammation and increased risk of thrombosis. Amin has observed a cumulative increase in pro-inflammatory markers at 6 h in swine forelimb perfusion [17]. Additionally, cellular blood-based perfusate is limited by blood bank accessibility, blood refrigeration, and the short shelf life of blood products, limiting its utility in military and emergency settings [63, 64]. Blood-based perfusates also carry risk of infection and coagulation, as well as HLA-sensitization and transfusion-related reactions [64–66].

By contrast, acellular media lacks cellular components and therefore generally relies on the dissolving of oxygen. Several studies in porcine lung EVMP suggest that acellular perfusates are a suitable alternative to blood-based perfusate [67–69]. Therefore, acellular perfusates have gained increasing interest as a more accessible and low-maintenance approach, evidenced by nearly half of the studies in this cohort using acellular perfusate. Importantly, while simpler and easier to manage, the absence of specialized oxygen carriers like RBCs may limit the efficiency of O_2_ transport. Thus, acellular media often need additional oxygenation such as adding synthetic oxygen carriers or pumping with oxygen [70].

### Base Medium

The base medium (see Table 4) can be roughly categorized into 3 different types: 1) cell culture, 2) electrolyte balance, 3) preservation and perfusion. They share many common functions, including basic functions like maintaining osmotic balance, cellular homeostasis, and regulation of pH. Some of the media contains nutrients like amino acids, glucose, or specialized carbohydrates, which can provide cells with additional substrates for metabolism support during preservation. Certain media like HTK has tryptophan which can protect the graft against oxidative stress during ischemic conditions [71].

### Supplements and Additives

There are a variety of supplements that can be added to tailor the perfusate to specific experimental conditions. Electrolytes are a common inclusion, especially sodium chloride, which is necessary to maintain the osmotic balance. Additionally, calcium and magnesium compounds serve important roles in cellular signaling and enzymatic functions. Potassium is important in maintaining a high intracellular-to-extracellular gradient via the Na + K + ATPase pump, as most total body potassium is stored within muscle.

The base media chosen also contains different additives that can help modulate the perfusate. Cell culture media like DMEM usually contain general nutritional components for cellular division. By contrast, STEEN and Perfadex include unique components like albumin and D_40_, which is specialized for specific organs like lungs. Fluosol-43 is designed to promote tissue oxygenation [72]. University of Wisconsin solution (UW) contains potassium lactobionate and raffinose, where the former compound is critical for minimizing cellular edema and the latter one is crucial in providing carbohydrate sources for metabolism. Custodiol HTK include histidine and tryptophan, amino acids that can help in maintaining pH balance and protecting cells during ischemic or hypothermic conditions.

### Perfusion Time

The duration of perfusion is a pivotal factor that may influence cellular viability, organ functionality, and the risk of ischemic injury. Even brief periods of ischemia can lead to significant tissue damage. Shorter perfusion times, generally around 6 h, are beneficial for minimizing logistical challenges and reducing the risk of complications. However, perfusion times ranging between 6 and 24 h can allow for better equilibration with the perfusion solution and potentially offer a broader window for assessing organ viability prior to transplant or replant. Extended perfusion durations that exceed 24 h are usually employed for experimental settings. While they allow for in-depth monitoring and potentially improved transplantation outcomes, these extended durations are logistically complex and pose an elevated risk of complications like delayed graft function. The decision regarding duration of perfusion requires thorough consideration of the aforementioned factors and should be tailored to the type of organ, logistical challenges, and overall objective of the perfusion.

### Limitations and Suggestions for Future Research

This systematic review presents with several limitations. Literature search was conducted with the assumption that all relevant studies would be discoverable via six large databases and a predetermined set of search terms. Additionally, non-English studies, abstracts, posters, conference presentations, and unpublished data were excluded from this study. In consideration of the small cohort of included studies, it is possible that we excluded other research that would offer valuable insight into the development of research in VCA EVMP. Specifically, the exclusion of non-English papers may have unintentionally limited this review, and further insights might be gleaned from supplementary examination of non-English VCA EVMP articles. Additionally, this review excludes articles published after June 2023. As VCA research is rapidly evolving, multiple studies may have been published on this topic in the intervening time.

The conclusions drawn from this review are limited by the quality and design of published research in VCA EVMP. As the swine forelimb represents the dominant model in this review, outcomes of these studies may not be generalizable to humans or other models with more complex forearm and hand anatomy. Future investigations in EVMP of monkey or ape limbs and subsequent functional testing may help to bridge this gap in knowledge. Additionally, the included studies are not representative of the breadth of VCA (e.g., face, calvarium, abdominal wall, and genital transplantation). As such, these studies may not be applicable to preservation of these structures.

While this paper details the technical aspects and limitations of VCA EVMP, these are not the only barriers to clinical translation. VCA is performed by a limited number of institutions, and on a significantly smaller scale than solid organ transplants. The low numbers of yearly VCAs are cost-prohibitive for a standardized perfusion machine, and severely limit the sample size for any potential clinical trials. VCAs also carry unique ethical considerations, including vulnerability of recipients, as well as racial and socioeconomic disparities [73]. These logistical and ethical barriers further hinder the successful clinical translation of EVMP in VCA.

## Conclusion

VCA EVMP is a versatile platform through which grafts may be preserved and optimized prior to replantation or replantation. There is significant evidence to suggest that EVMP may be superior to SCS as a preservation method. While methods greatly varied throughout the literature reviewed, the major factors of each perfusion protocol remained the same: temperature, perfusate composition, and perfusion time. As in solid organ transplant perfusion, there is currently no consensus on the optimal temperature for VCA perfusion. Studies reviewed in this paper showed promising results for both HMP and NMP/NNT, and no recent evidence has definitively suggested the benefit of one temperature over the other. Rather than attempting to condense VCA EVMP down to a singular optimal perfusion protocol, perfusion factors should be chosen and adapted based on the individual needs and goals of each future study. For instance, the choice of a blood-based perfusate might be more suitable for NMP given the higher metabolic rate, or for a shorter perfusion duration given the limitations of obtaining and storing blood. An acellular perfusate might be more suitable for HMP given the lower metabolic rate, or for a longer perfusion duration to facilitate perfusate exchange. Overall, preclinical studies offer promising results regarding the feasibility of VCA preservation via machine perfusion, but additional experimental studies are needed to overcome technical barriers to clinical translation.

## Data Availability

The original contributions presented in the study are included in the article/supplementary material, further inquiries can be directed to the corresponding author.
